# ReishiMax inhibits mTORC1/2 by activating AMPK and inhibiting IGFR/PI3K/Rheb in tumor cells

**DOI:** 10.1038/s41392-019-0056-7

**Published:** 2019-06-28

**Authors:** Didem Sohretoglu, Chao Zhang, Jun Luo, Shile Huang

**Affiliations:** 1 Department of Pharmacognosy, Faculty of Pharmacy, Hacettepe University, TR 06100 Ankara, Turkey; 2 Department of Biochemistry and Molecular Biology, Louisiana State University Health Sciences Center, 1501 Kings Highway, Shreveport, LA 71130-3932 USA; 3 Key Laboratory of National Health and Family Planning Commission on Parasitic Disease Control and Prevention, Jiangsu Provincial Key Laboratory on Parasite and Vector Control Technology, Jiangsu Institute of Parasitic Diseases, 214064 Wuxi, Jiangsu Province China; 4 College of Veterinary Medicine, South China Agricultural University, 510642 Guangzhou, China; 5 Feist-Weiller Cancer Center, Louisiana State University Health Sciences Center, 1501 Kings Highway, Shreveport, LA 71130-3932 USA

**Keywords:** Target identification, Cancer therapy

## Abstract

*Ganoderma lucidum* (*G. lucidum*) extracts, as dietary supplements, have been found to exert potent anticancer activity, which is attributed to the presence of polysaccharides and triterpenes. However, the molecular mechanism underlying the anticancer action of *G. lucidum* extracts remains to be investigated. Here, we show that ReishiMax GLp, containing *G. lucidum* polysaccharides and triterpenes (GLPT), inhibited cell proliferation and induced cell death in human lung cancer cells (A549 and A427) and simultaneously suppressed the signaling pathways of mammalian target of rapamycin complexes 1 and 2 (mTORC1 and mTORC2), respectively. Mechanistically, GLPT downregulated the phosphorylation and protein levels of insulin-like growth factor 1 receptor (IGFR) and phosphoinositide 3-kinase (PI3K) as well as the protein level of RAS homolog enriched in brain (Rheb). In addition, GLPT also activated the AMP-activated protein kinase (AMPK) network. This was evidenced by observations that GLPT increased the phosphorylation of AMPKα (T172) and its substrates tuberous sclerosis complex 2 (TSC2, S1387) and regulatory-associated protein of mTOR (raptor, S792). Ectopic expression of dominant-negative AMPKα partially mitigated the inhibitory effect of GLPT on mTORC1, indicating that GLPT inhibits mTORC1 partly by activating AMPK. The results suggest that *G. lucidum* extracts exert anticancer action at least partly by suppressing mTORC1/2 signaling via activation of AMPK and inhibition of IGFR/PI3K/Rheb in tumor cells.

## Introduction

The medicinal mushroom *Ganoderma lucidum* (*G. lucidum*) has been traditionally served as a dietary supplement in Oriental countries to promote health and longevity for a long time.^1^ Evidence has shown that *G. lucidum* exerts a variety of biological activities, including anti-inflammatory, antioxidant, antiglycemic, antiulcer, anticancer, and immunostimulatory effects.^1,2^ Of note, *G. lucidum* executes its anticancer activity mainly via its polysaccharides (from water-soluble extracts) and triterpenes (from water-insoluble extracts).^1,2^ *G. lucidum* and its extracts have been documented as potential anticancer agents for various tumors, including those in melanoma,^3,4^ leukemia, lymphoma, myeloma,^5,6^ breast cancer,^4–7^ prostate cancer,^4–8^ ovarian cancer,^9^ bladder cancer,^10^ head and neck cancer,^11^ lung cancer,^12–14^ liver cancer, ^15,16^ gastric cancer,^17^ and colon cancer.^18,19^

*G. lucidum* extracts containing both polysaccharides and triterpenes can directly inhibit cell proliferation, induce cell death and suppress the migration/invasion of tumor cells in vitro and inhibit tumor growth and metastasis in vivo.^1,2^ Studies have reported the various molecular mechanisms underlying these actions, including downregulation of c-myc,^20,21^ cyclin D1/E/B1,^8,9,21–24^ cyclin-dependent kinases (CDKs), ^14,23–25^ survivin,^26^ vascular endothelial growth factor (VEGF),^27,28^ and matrix metalloproteinase 2/9 (MMP-2/9);^29,30^ upregulation of CDK inhibitors (p21^Cip1^ and p27^Kip1^);^8,22,24^ inhibition of focal adhesion kinase (FAK),^31^ small GTPases,^31^ nuclear factor kappa B (NF-κB),^25,32^ protein kinase C (PKC),^15^ and Akt;^14,33–35^ and activation of p38 and c-Jun N-terminal kinase (JNK).^15,21^ While it is probable that *G. lucidum* extracts may impact each of these individual signaling molecules depending on the cell types and/or experimental conditions, it seems more conceivable that *G. lucidum* extracts may target certain major targets directly, subsequently influencing the abovementioned targets indirectly.

mTOR (mammalian target of rapamycin) is recognized as a hub that regulates cell growth, survival, and metabolism.^36,37^ Deregulated mTOR signaling has been frequently observed in various types of tumors, so mTOR is regarded as a promising target for cancer therapy.^36^ Current knowledge indicates that mTOR functions as two mTOR complexes (mTORC1 and mTORC2) in mammalian cells.^36^ mTORC1 senses insulin/growth factors, amino acids, energy, oxygen, and DNA damage, while mTORC2 primarily senses insulin/growth factors.^36^ Both mTORC1 and mTORC2 can be positively regulated by the IGFR-PI3K (insulin-like growth factor-1 (IGF-1) receptor-phosphatidylinositol 3′ kinase) pathway, which is antagonized by PTEN (phosphatase and tensin homolog).^36^ In addition, mTORC1 is negatively regulated by AMPK (AMP-activated protein kinase).^38^ Low energy levels, oxidative stress or hypoxia activates AMPK, which can phosphorylate TSC2 (tuberous sclerosis complex 2) at multiple sites (including S1387), leading to activation of the TSC1/2 complex.^38,39^ The activated TSC complex antagonizes Rheb (RAS homolog enriched in brain) by hydrolyzing GTP-Rheb into GDP-Rheb, thereby inhibiting Rheb-mediated effects on mTORC1.^39,40^ In addition, activated AMPK can also phosphorylate regulatory-associated protein of mTOR (raptor) on S792, leading to inhibition of mTORC1.^36^ While S6K1 (p70 S6 kinase 1) and 4E-BP1 (eukaryotic initiation factor 4E binding protein 1) are two well-known substrates of mTORC1, Akt (S473) is the best-characterized substrate of mTORC2.^36^ Although the biological functions of mTORC1/2 remain to be further determined, evidence indicates that mTOR can control the expression/activity of c-myc, cyclin D1, cyclin-dependent kinases (CDKs), the CDK inhibitor p27^Kip1^, VEGF, survivin, JNK, NF-κB, and MMP-2.^42^ Interestingly, of the signaling molecules mediated by mTOR, many of them, e.g., c-myc, cyclin D1, CDKs, p27^Kip1^, survivin, NF-κB, JNK, FAK, small GTPases, MMP-2, and VEGF, are also targeted by *G. lucidum* extracts.^20–35^ Thus, we hypothesized that *G. lucidum* extracts may exert anticancer effects primarily by targeting mTOR signaling. This study was designed to test this hypothesis using human lung cancer cells (A549 and A427 cells) as experimental models.

## Results

### GLPT inhibits cell proliferation and induces cell death in lung cancer cells

**Fig. 1:**
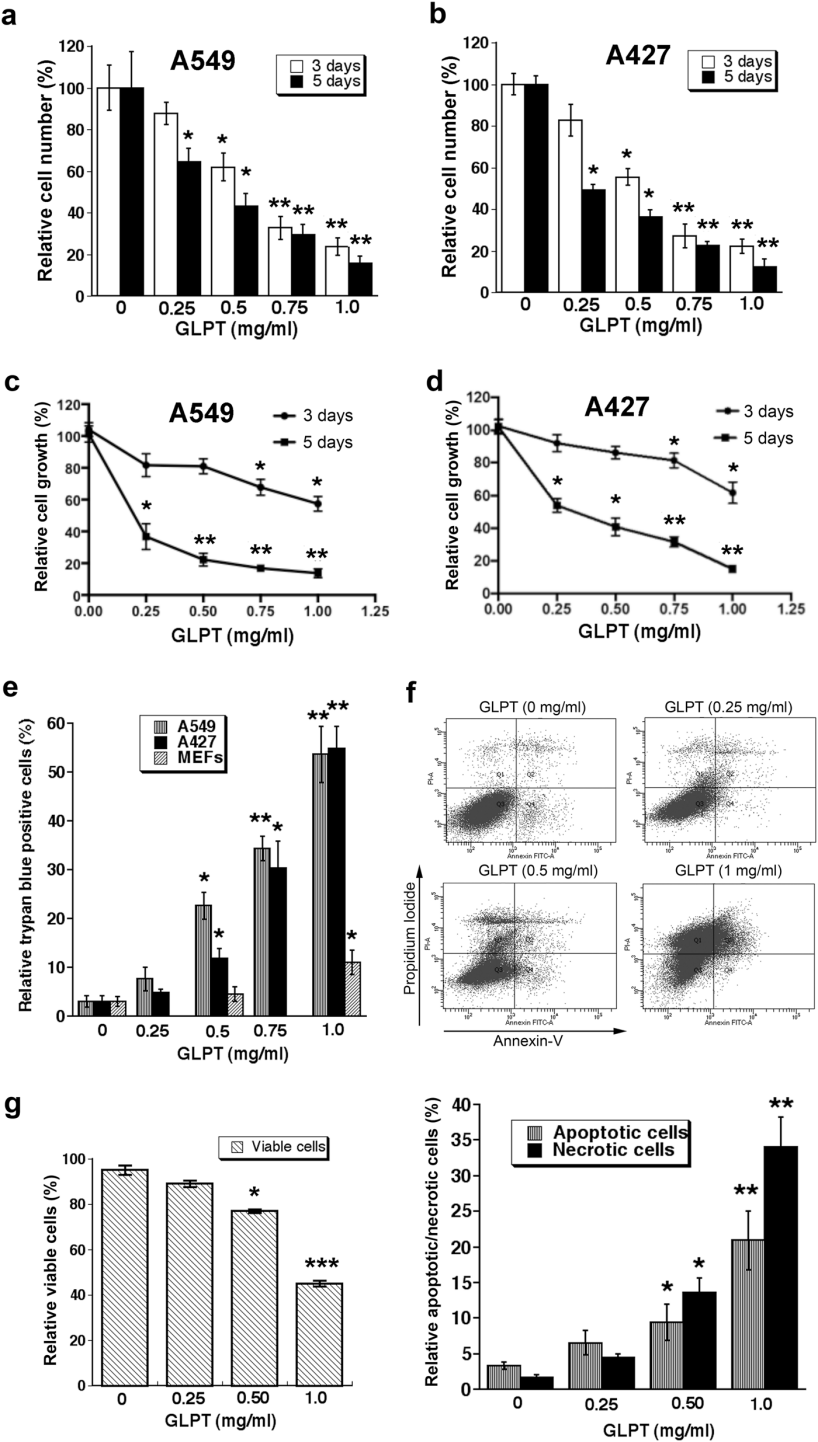
GLPT inhibits cell proliferation and induces cell death in lung cancer cells. **a–d** A549 and A427 cells were treated with GLPT (0–1 mg/ml) for 3 or 5 days and then subjected to cell counting (**a**, **b**) or one-solution assay (**c**, **d**). **e–g** The indicated cells were treated with GLPT (0–1 mg/ml) for 72 h and then analyzed with trypan blue exclusion assay (**e**) or with annexin V-PI staining followed by flow cytometry (**f**, **g**). Representative images of the flow cytometry results are shown in (**f**), and the quantitative data are presented in (**g**). All data represent the mean ± SD (*n* = 3). GLPT treatment *versus* control (0 mg/ml GLPT), *P < 0.05; **P < 0.01; ***P < 0.001

It is known that *G. lucidum* executes its antitumor activity primarily via the joint action of triterpenes and polysaccharides [23]. To better evaluate the antitumor activity of *G. lucidum*, we employed ReishiMax (GLPT), a *G. lucidum* extract containing 13.5% polysaccharides and 6% triterpenes [24]. First, to assess the antiproliferative effect of GLPT on tumor cells, human A549 and A427 lung cancer cells were incubated with GLPT (0–1 mg/ml) for 3 or 5 days and then enumerated. The results showed that 5-day treatment with GLPT dose-dependently suppressed cell proliferation (Fig. 1a, b), with IC_50_ values of 0.39 mg/ml (A549) and 0.32 mg/ml (A427). Similar data were obtained in one-solution assays (Fig. 1c, d). These results indicate that GLPT inhibits cancer cell proliferation.

In addition, we also tested the cytotoxic effect of GLPT on A549 and A427 cells. Treatment with GLPT for 72 h increased the number of trypan blue-positive cells dose-dependently (Fig. 1e). Compared to the vehicle (DMSO), 0.5 mg/ml GLPT induced ~4-fold and 10-fold more cell death in A427 and A549 cells, respectively, whereas 1.0 mg/ml GLPT was able to increase cell death by nearly 20-fold in both A427 and A549 cells. Interestingly, at 0.5 mg/ml, GLPT did not exhibit obvious cytotoxic effects on normal (i.e., nontumorous) mouse embryonic fibroblasts (MEFs); at 1.0 mg/ml, GLPT induced massive (~55%) cell death in A427 and A549 cells but only induced modest (11%) cell death in MEFs (Fig. 1e). Collectively, these data suggest that GLPT has potent cytotoxic effects on cancer cells rather than normal cells.

The polysaccharides and triterpenes of *G. lucidum* have been reported to induce apoptosis in a variety of tumor cells.^1,2^ Having observed that treatment with GLPT for 72 h is able to induce concentration-dependent cell death in A549 and A427 cells (Fig. 1e), we reasoned that GLPT might also induce apoptosis in lung cancer cells. To this end, A427 cells were treated with 0–2 mg/ml GLPT. After treatment for 72 h, the cells were stained with Annexin V-PI (propidium iodide) and then subjected to flow cytometry, a conventional assay for apoptosis and necrosis. The results showed that treatment with 0.5 and 1.0 mg/ml GLPT for 72 h significantly reduced the percentages of viable cells from 95.2% (in cells treated with DMSO) to 77.0% and 45.1%, respectively (Fig. 1f, g). Consistent with this finding, treatment with GLPT increased cell death (apoptosis and necrosis) dose-dependently. GLPT at 0.5 and 1.0 mg/ml increased the percentages of apoptotic cells from 3.3% (vehicle control) to 9.4% and 20.9%, respectively, and increased the percentages of necrotic cells from 1.6% (vehicle control) to 13.6% and 34.0%, respectively. The results reveal that GLPT can induce both apoptosis and necrosis in lung cancer cells.

### GLPT induces G_1_ cell cycle arrest in lung cancer cells

**Fig. 2:**
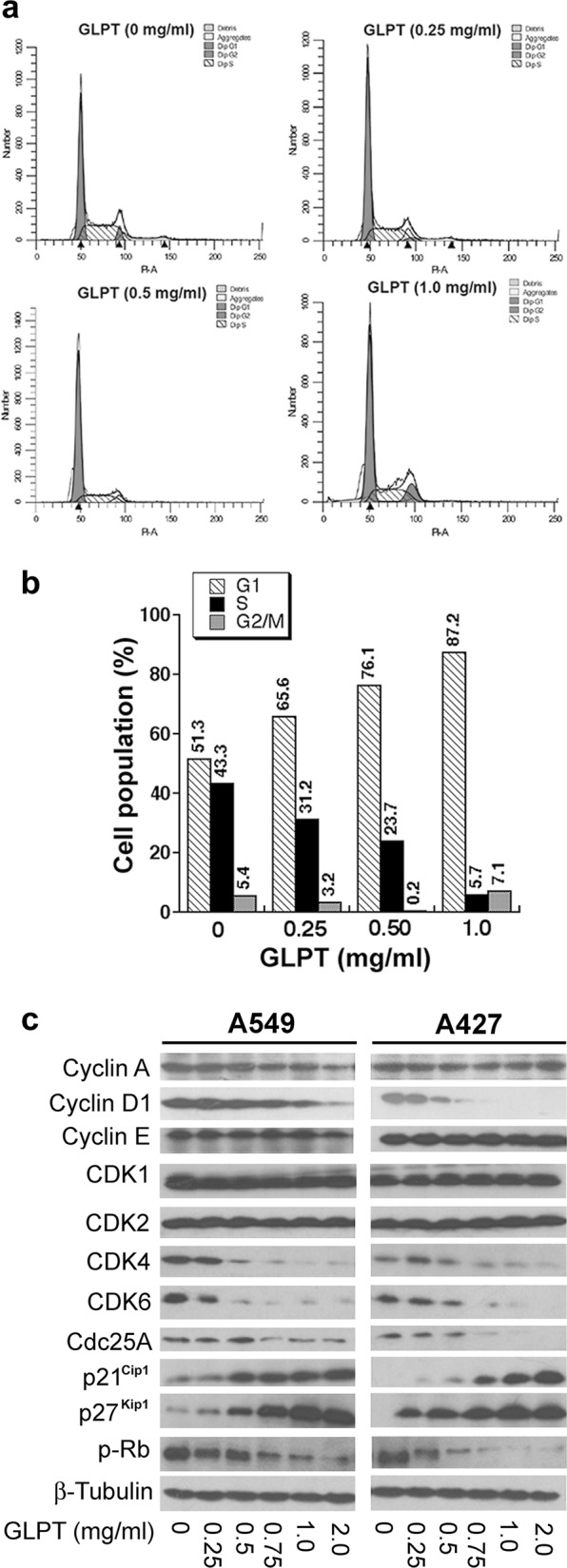
GLPT arrests lung cancer cells in the G_1_ phase of the cell cycle by downregulating the protein levels of cyclin D1, Cdc25A, and CDK4/6 and upregulating the protein levels of p21^Cip1^ and p27^Kip1^, leading to hypophosphorylation of Rb. **a**, **b** A549 cells were treated with GLPT (0–1 mg/ml) for 24 h and then subjected to cell cycle analysis. Representative images of the flow cytometry results are presented in (**a**), and the quantitative data for (**a**) are shown in **b**. The bar graphs show the effect of GLPT on the proportion (%) of A549 cells in the G_1_, S, and G_2_/M phases of the cell cycle. Similar results were observed in three independent experiments. **c** A549 and A427 cells were treated with GLPT for 24 h at the indicated concentrations, and western blotting was then performed with the indicated antibodies. β-Tubulin served as a loading control

Cell proliferation and cell division are closely associated with cell cycle progression.^43^ To elucidate how GLPT inhibits lung cancer cell proliferation, cell cycle analysis was conducted. As the doubling time of the A549 cell line is ~22 h (ATCC information sheet), the cells were treated with GLPT for 24 h. We found that such treatment increased the cell population in the G_0_/G_1_ phase in a dose-dependent manner. GLPT at 0.25, 0.5 and 1.0 mg/ml significantly increased the proportion of cells in G_0_/G_1_ phase from 51.3% (control) to 65.6%, 76.1% and 87.2%, respectively (Fig. 2a, b). These data suggest that GLPT inhibits lung cancer cell proliferation by inducing G_1_ cell cycle arrest.

Cell cycle progression is tightly regulated by the coordinated activities of cyclin/CDK complexes, CDK inhibitors and Cdc25.^43^ To elucidate how GLPT induces G_1_ cell cycle arrest in lung cancer cells, we determined the levels of proteins related to G_1_ phase-to-S phase progression. Our western blot analysis showed that GLPT treatment (24 h) dose-dependently reduced the cellular protein levels of cyclin D1, CDK4, CDK6, and Cdc25A and increased the protein levels of the CDK inhibitors p21^Cip1^ and p27^Kip1^ (Fig. 2c). Notably, CDK1 and CDK2 were not obviously affected by GLPT treatment, despite a slight inhibitory effect of 2 mg/ml GLPT on cyclin A/E expression in A549 cells (Fig. 2c). Rb (retinoblastoma protein) acts as a checkpoint protein regulating cell cycle progression from G_1_ phase to S phase.^43^ As predicted, GLPT treatment for 24 h also inhibited Rb phosphorylation dose-dependently (Fig. 2c). These observations demonstrate that GLPT induces G_1_ cell cycle arrest associated with inhibition of G_1_ cyclins/CDKs, leading to decreased phosphorylation of Rb.

### GLPT induces caspase-dependent apoptosis in lung cancer cells

**Fig. 3:**
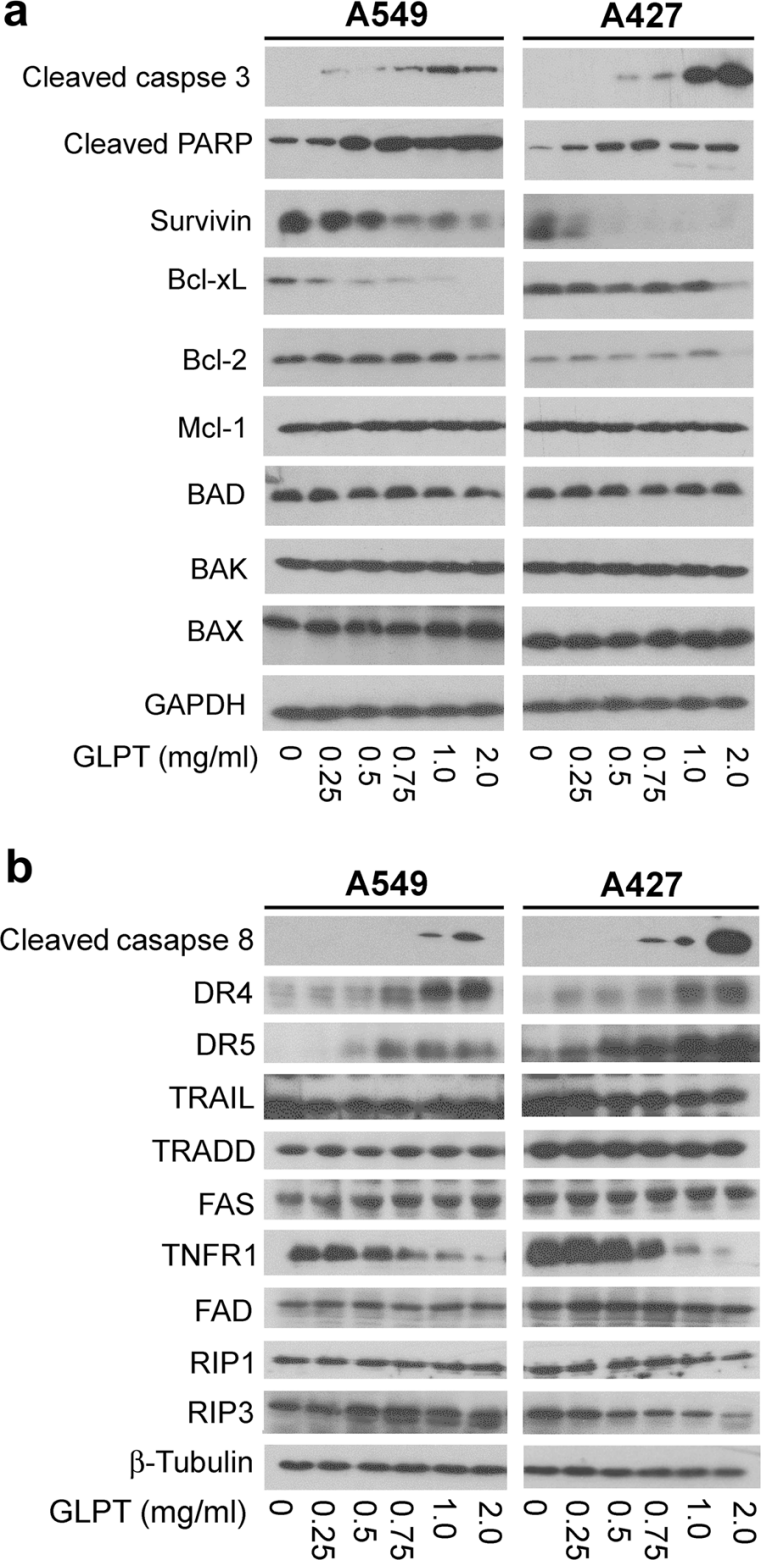
GLPT downregulates the expression of the antiapoptotic proteins Bcl-xL and survivin and upregulates the expression of DR4 and DR5, leading to activation of caspase 3/8 and cleavage of PARP. **a**, **b** A549 and A427 cells were treated with GLPT for 24 h at the indicated concentrations, and western blotting was then performed with the indicated antibodies. GAPDH or β-tubulin served as a loading control

Caspase-dependent and caspase-independent apoptosis can be induced under different conditions.^44^ Having observed GLPT-induced apoptosis in lung cancer cells (Fig. 1f, g), we wondered whether this apoptosis was caspase-dependent. To answer this question, cleaved PARP, which is a hallmark of caspase-dependent apoptosis,^44^ was probed by western blotting. The results showed that 24-h treatment with GLPT increased the level of cleaved PARP dose-dependently in both A549 and A427 cells (Fig. 3a), indicating caspase-dependent apoptosis. This finding was further supported by the observation that GLPT activated caspase 3, as judged by increased cleavage of caspase 3 in A549 and A427 cells revealed by western blot analysis (Fig. 3a).

Caspase-dependent apoptosis can be mediated by the mitochondrial and death receptor pathways.^44,45^ To determine the pathways involved in the observed apoptosis, we first investigated whether GLPT-induced apoptosis occurs through the mitochondrial pathway. Since the Bcl-2 family proteins are the key players in this pathway, the levels of these proteins were tested. We found that treatment with GLPT (0–2 mg/ml) for 24 h did not apparently affect the levels of the proapoptotic proteins BAD and BAK, despite a marginal increase in BAX in A549 and A427 cells. In contrast, GLPT treatment greatly decreased the levels of the antiapoptotic proteins survivin and Bcl-xL in a dose-dependent manner (Fig. 3a). At 2 mg/ml, GLPT also reduced the level of Bcl-2. The results indicate that GLPT-induced apoptosis is partly mediated by activation of the mitochondrial pathway mainly associated with downregulation of survivin, Bcl-xL and Bcl-2 (antiapoptotic proteins).

Next, we further studied whether the death receptor pathway is involved in GLPT-induced apoptosis. Since caspase 8 is a crucial initiator caspase for the activation of the death receptor pathway,^45^ we first examined whether GLPT activates caspase 8. Interestingly, treatment with GLPT (0–2 mg/ml) for 24 h increased the level of cleaved caspase 8 (Fig. 3b), indicating activation of caspase 8.

To better understand how the death receptor pathway was activated, we investigated whether GLPT affects the levels of related ligands, death receptors, and adaptor proteins. Treatment with GLPT (0–2 mg/ml) for 24 h did not obviously influence the expression of TRAIL, TRADD, FASL, FADD, and RIP1 but drastically upregulated the protein levels of DR4 and DR5 (Fig. 3b). Surprisingly, the protein level of TNFR1 was profoundly reduced by GLPT in a dose-dependent manner. In addition, in response to GLPT treatment, RIP3, which is involved in the regulation of necroptosis,^46^ was markedly upregulated in A549 cells but downregulated in A427 cells. The above results suggest that GLPT-induced apoptosis is also in part linked to increased expression of DR4 and DR5 in lung cancer cells. Taken together, our findings reveal that GLPT is able to induce caspase-dependent apoptosis through both the mitochondrial and death receptor pathways.

### GLPT inhibits both the mTORC1 and mTORC2 pathways

**Fig. 4:**
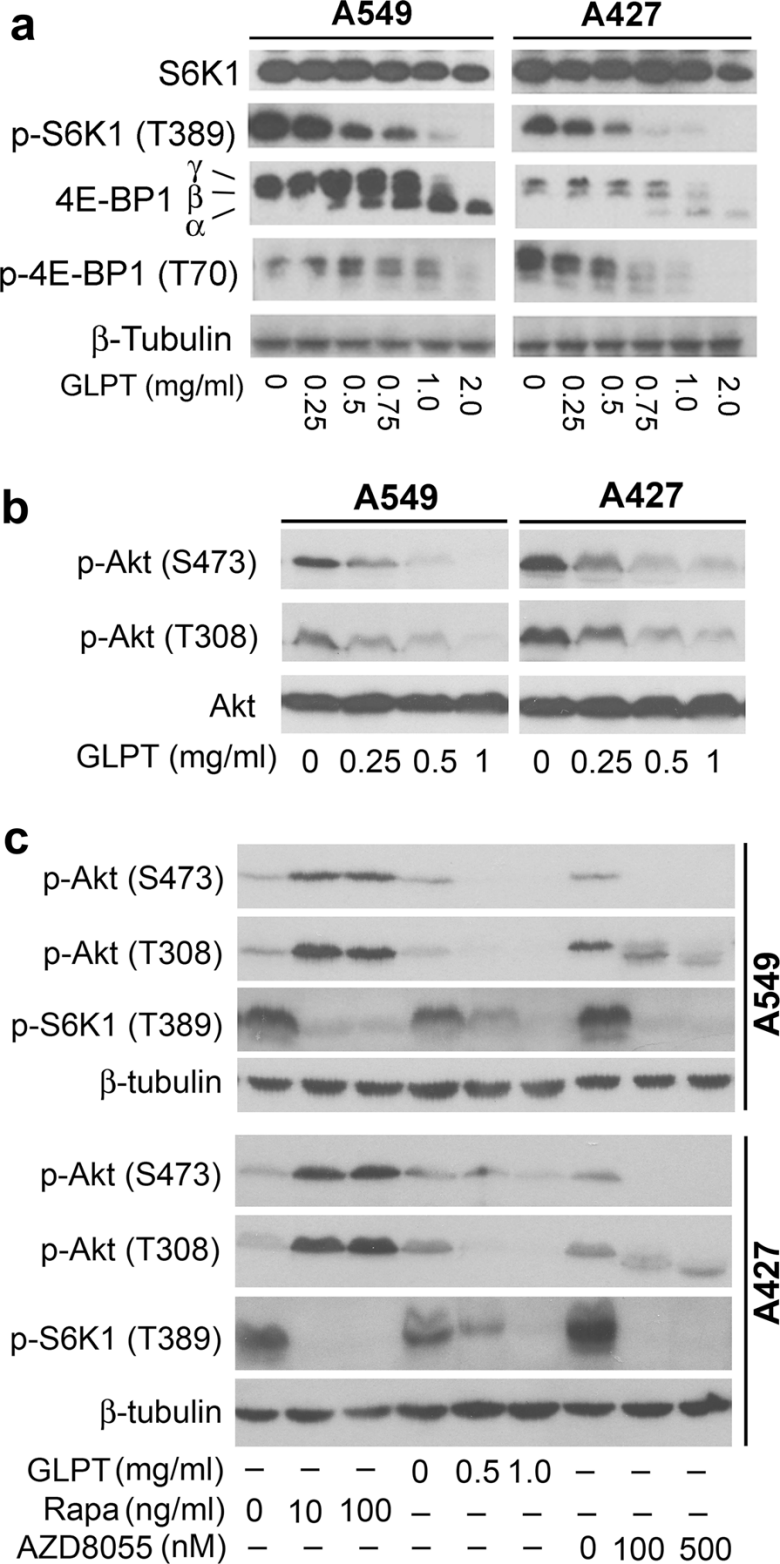
GLPT inhibits mTORC1/2 signaling pathways. **a**–**c** A549 and A427 cells were treated with GLPT for 24 h, rapamycin (Rapa) for 2 h or AZD8055 for 2 h at the indicated concentrations, and western blotting was then performed with the indicated antibodies. β-Tubulin served as a loading control. Similar results were observed in at least three independent experiments

mTOR plays a central role in the regulation of cell proliferation and survival.^36^ To determine whether *G. lucidum* might execute its anticancer action by inhibiting the mTOR pathway, A549 and A427 cells were treated with GLPT (0–2 mg/ml) for 24 h, and then western blot analysis of mTOR signaling molecules was performed. We observed that GLPT dose-dependently inhibited the phosphorylation of S6K1 (T389), a well-known substrate of mTORC1,^36^ and remarkable inhibition started at 0.5 mg/ml (Fig. 4a). Similarly, GLPT also inhibited the phosphorylation of 4E-BP1, another major substrate of mTORC1,^36^ in the cells. In addition, treatment with GLPT for 24 h also dose-dependently decreased the phosphorylation of Akt (S473) (Fig. 4b), a substrate of mTORC2.^36^ Similarly, GLPT also inhibited the phosphorylation of Akt (T308) (Fig. 4b), which is mediated by PDK1 (phosphoinositide-dependent kinase 1).^36^ One control, rapamycin, an allosteric inhibitor of mTORC1, inhibited mTORC1-mediated phosphorylation of S6K1 (T389) but increased mTORC2-mediated phosphorylation of Akt (S473) (Fig. 4c); another control, AZD8055, an ATP-competitive and selective inhibitor of mTOR, inhibited both mTORC1 and mTORC2 (Fig. 4c). Collectively, the results indicate that GLPT inhibits both the mTORC1 and mTORC2 signaling pathways in lung cancer cells.

### GLPT inhibition of mTORC1/2 is related to activation of AMPK and inhibition of IGFR/PI3K/Rheb

**Fig. 5:**
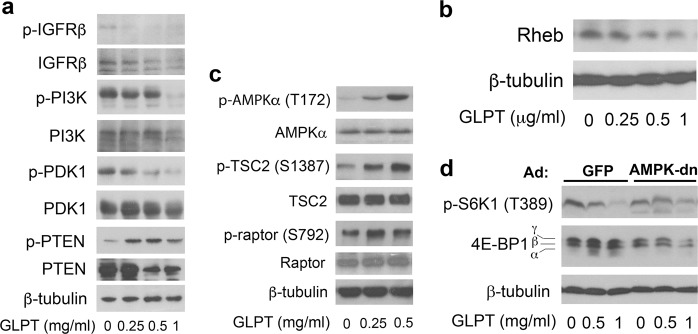
GLPT-mediated inhibition of mTORC1/2 is associated with activation of AMPK and inhibition of IGFR/PI3K/Rheb. **a**–**d** A549 cells (**a**–**c**) or A549 cells infected with Ad-AMPK-dn or Ad-GFP (control) (**d**) were treated with GLPT for 24 h at the indicated concentrations, and western blotting was then performed with the indicated antibodies. β-Tubulin served as a loading control

Since both mTORC1 and mTORC2 are positively mediated by the IGFR-PI3K pathway and negatively regulated by PTEN,^36,42^ to understand how GLPT inhibits mTORC1/2, we next investigated whether GLPT inhibits mTORC1/2 by altering the expression/activity of these signaling proteins. As predicted, treatment with GLPT for 24 h dose-dependently reduced the levels of p-IGFRβ, p-PI3K (p85), and p-PDK1 in A549 cells (Fig. 5a). Additionally, GLPT dose-dependently downregulated the protein level of IGFRβ but did not reduce the protein levels of PI3K (p85) and PDK1 except at concentrations of 1 mg/ml and higher. Unexpectedly, GLPT treatment inhibited rather than activated PTEN, as indicated by the observation that GLPT decreased the protein level and increased the phosphorylation level of PTEN (Fig. 5a). Thus, the results suggest that GLPT inhibition of mTORC1/2 is related to downregulation of the IGFR-PI3K pathway but not to upregulation of PTEN.

As mTORC1 is also negatively regulated by the AMPK-TSC2/raptor network and positively regulated by Rheb,^38–41^ we also examined whether GLPT suppresses mTORC1 by activating AMPK and inhibiting Rheb. As expected, 24-h treatment with GLPT dose-dependently downregulated the protein level of Rheb (Fig. 5b), a direct activator of mTORC1.^39,40^ In addition, GLPT treatment also activated the AMPK network, as evidenced by increased phosphorylation of AMPKα (T172) and the two substrates of AMPK, TSC2 (S1387) and raptor (S792), in A549 cells (Fig. 5c). Interestingly, ectopic expression of dominant-negative AMPKα (AMPKα-dn) by infection with Ad-AMPKα-dn partially prevented GLPT from inhibiting mTORC1-mediated S6K1 and 4E-BP1 phosphorylation (Fig. 5d), indicating that GLPT indeed inhibits mTORC1 partly through activation of AMPK. Collectively, these results suggest that GLPT inhibits mTORC1 in part by activating AMPK and inhibiting Rheb in addition to suppressing the IGFR-PI3K pathway.

## Discussion

Numerous studies have shown that *G. lucidum* extracts exert anticancer effects.^3–19^ However, the mechanism behind the anticancer action is not well understood. mTOR plays a pivotal role in controlling cell proliferation and survival.^36^ Here, we found that GLPT, a standardized *G. lucidum* extract containing both polysaccharides and triterpenes, inhibited cell proliferation and induced cell death and concurrently suppressed mTORC1-mediated phosphorylation of S6K1 and 4E-BP1 and mTORC2-mediated phosphorylation of Akt in human lung cancer cells (A549 and A427 cells). Our results are, to some degree, in agreement with some previous findings.^14,33,34^ For instance, treatment with *G. lucidum* polysaccharides inhibits the phosphorylation of 4E-BP1 and ribosomal protein S6 (a substrate of S6K1) in SUM-149 breast cancer cells.^34^ Additionally, treatment with active lipids of *G. lucidum* spores reduces the phosphorylation levels of ERK1/2 and Akt and increases the phosphorylation level of JNK1/2 in THP-1 leukemia cells.^33^ Together, these findings suggest that inhibition of mTORC1/2 pathways could be one of the main mechanisms underlying the anticancer action of *G. lucidum* extracts.

In this study, we found that GLPT inhibited the proliferation of A549 and A427 lung cancer cells by inducing cell cycle arrest in G_1_ phase. This arrest was achieved through reductions in the levels of G_1_/S proteins, e.g., CDK4, CDK6, cyclin D1, and Cdc25A, and increases in the levels of p27^Kip1^ and p21^Cip1^ (CDK inhibitors), resulting in hypophosphorylation of Rb (Fig. 2). In addition, we also noticed that GLPT induced caspase-dependent apoptosis through both the mitochondrial and death receptor pathways. This effect was evidenced by observations that GLPT increased the levels of cleaved caspase 3/8 and cleaved PARP by reducing the levels of antiapoptotic proteins (Bcl-xL, survivin, and Bcl-2) and increasing the levels of DR4 and DR5 (death receptors) in the cells (Fig. 3). Previous studies on other cell lines have shown that *G. lucidum* extracts exert anticancer effects by downregulating the expression/activity of c-myc, cyclin D1/B1, CDKs, survivin, NF-κB, FAK, small GTPases, MMP-2, and VEGF and upregulating the expression/activity of p27^Kip1^ and JNK.^20–35^ mTOR is known as a central controller of cell growth/proliferation, motility, and survival.^36,42^ It has been demonstrated that mTOR mediates the expression/activity of c-myc, cyclin D1, CDKs, p27^Kip1^, VEGF, survivin, JNK, MMP-2, and NF-κB.^42^ This study revealed that GLPT inhibited both mTORC1 and mTORC2 signaling (Fig. 4). In view of our data and that of others,^20–35^ we here tentatively propose that *G. lucidum* extracts might execute anticancer activity primarily by inhibiting mTOR, indirectly resulting in the up/downregulation of many other signaling molecules. Undoubtedly, further research is needed to confirm this viewpoint.

It is known that rapamycin, the first described allosteric mTOR inhibitor, inhibits mTORC1 but activates mTORC2-mediated phosphorylation of Akt (S473) through a negative feedback mechanism, while AZD8055 (an mTOR kinase inhibitor) inhibits both mTORC1 and mTORC2.^36,42^ This study found that GLPT, similar to AZD8055, inhibited both mTORC1-mediated S6K1 and 4E-BP1 phosphorylation and mTORC2-mediated Akt (S473) phosphorylation (Fig. 4c). However, unlike AZD8055 or rapamycin, GLPT was able to induce AMPKα (T172) phosphorylation and subsequently induce AMPK-mediated TSC2 (S1387)/raptor (S792) phosphorylation (Fig. 5c). Expression of dn-AMPKα partially lessened the inhibitory effect of GLPT on mTORC1 (Fig. 5d), indicating that GLPT inhibits mTORC1 partly via activation of AMPK. In addition, GLPT also reduced the protein level of Rheb (Fig. 5b), a direct activator of mTORC1.^39,40^ Moreover, GLPT was also able to suppress the IGFR*-*PI3K pathway in the cancer cells (Fig. 5a). Taken together, our data demonstrate that GLPT-mediated inhibition of mTORC1/2 is a consequence of inhibition of the IGFR-PI3K pathway, activation of the AMPK-TSC2/raptor network and downregulation of the protein expression of Rheb. Therefore, our findings suggest that GLPT may be a novel mTOR inhibitor. How GLPT activates AMPK and inhibits IGFR/PI3K/Rheb to cause the inhibition of mTORC1/2 remains unclear. Studies have shown that increased levels of reactive oxygen species (ROS) or intracellular calcium can modify the structures and functions of cellular proteins, resulting in their inhibition or activation.^47^ It has been described that *G. lucidum* extracts can increase intracellular calcium levels and induce ROS production in MCF-7 breast cancer cells.^48^ Hence, it will be interesting to determine whether GLPT inhibits mTORC1/2 signaling by increasing the intracellular levels of ROS, calcium or both.

**Fig. 6:**
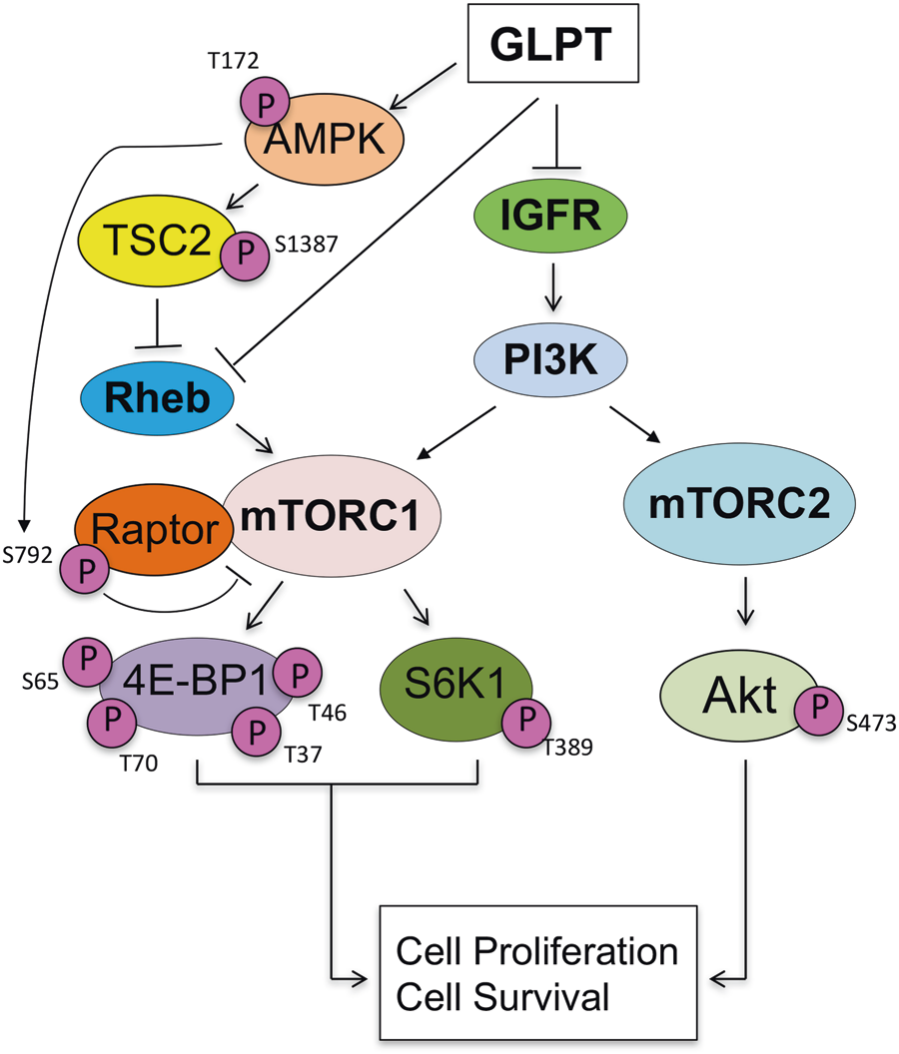
A diagram showing that GLPT inhibits mTORC1/2-mediated signaling pathways, thereby inhibiting cell proliferation and inducing cell death in tumor cells

In conclusion, we have demonstrated that GLPT (*G. lucidum* extracts) inhibited cell proliferation by decreasing the protein levels of CDK4/6, cyclin D1, and Cdc25A; increasing the protein levels of p27^Kip1^ and p21^Cip1^; inducing apoptosis by downregulating Bcl-xL, survivin and Bcl-2; and upregulating DR4 and DR5 in human lung cancer cells. Additionally, GLPT not only inhibited mTORC1-mediated S6K1 and 4E-BP1 phosphorylation but also repressed mTORC2-mediated Akt phosphorylation. This effect was associated with inhibition of the IGFR-PI3K pathway, activation of the AMPK-TSC2/raptor network, and downregulation of the protein expression of Rheb (Fig. 6). The results support the notion that inhibition of mTORC1/2 pathways may be one of the major mechanisms underlying the anticancer action of *G. lucidum* extracts.

## Materials and methods

### Materials

ReishiMax GLp^TM^, a standardized *G. lucidum* extract containing 13.5% polysaccharides and 6% triterpenes^20^ here termed GLPT, was purchased from Pharmanex (Provo, UT, USA). The GLPT was dissolved in DMSO (dimethyl sulfoxide) to prepare a fresh 50 mg/ml stock and then diluted to the necessary concentrations with medium before use. Rapamycin and AZD8055 were purchased from LC Laboratories (Woburn, MA, USA) and ApexBio (Boston, MA, USA), respectively. MEM nonessential amino acids, DMEM (Dulbecco’s modified Eagle’s medium), and 0.05% trypsin-EDTA were provided by Mediatech (Manassas, VA, USA). FBS (fetal bovine serum) was obtained from Atlanta Biologicals (Lawrenceville, GA, USA). A CellTiter 96^®^ Aqueous Non-Radioactive Cell Proliferation Assay Kit was purchased from Promega (Madison, WI, USA). A Cellular DNA Flow Cytometric Analysis Kit and an Annexin V-FITC Apoptosis Detection Kit I were obtained from Roche Diagnostics (Indianapolis, IN, USA) and BD Biosciences (San Jose, CA, USA), respectively. The antibodies used in the study included CDK1, cyclin E, TNFR1, FasL, TRAIL, DR4, DR5, TRADD, FADD, RIP1, RIP3, PI3K p85α/β/γ, p-IGFRβ (Tyr1161), IGFRβ (Santa Cruz Biotechnology, Santa Cruz, CA, USA), β-tubulin (Sigma, St Louis, MO, USA), cleaved PARP, cleaved caspase 8, cleaved caspase 3, p-S6K1 (T389), S6K1, p-4E-BP1 (T70), 4E-BP1, p-Akt (S473), p-Akt (T308), Akt, p-PDK1 (S241), PDK1, p-AMPKα (Thr172), p-raptor (S792), p-TSC2 (S1387), p-PTEN (Ser380/Thr382/383), PTEN, p-PI3K p85 (Tyr458)/p55 (Tyr199), Rheb, and GAPDH (Cell Signaling, Danvers, MA, USA) antibodies. All other antibodies and reagents were obtained as previously described.^49^

### Cell lines and culture

The human lung adenocarcinoma cell lines A549 and A427 were obtained from the ATCC (American Type Culture Collection) (Manassas, VA, USA). Wild-type mouse embryonic fibroblasts (MEFs) were generously provided by Charles J. Sherr (St. Jude Children’s Research Hospital, Memphis, TN, USA). All cells were cultured in DMEM supplemented with 10% FBS and 1% MEM nonessential amino acids in a humid incubator (37 °C and 5% CO_2_) and trypsinized with 0.05% trypsin-EDTA for subculture or experiments.

### Analyses of cell viability and proliferation

Cell viability was assessed with trypan blue exclusion assay, and cell proliferation was evaluated with one-solution assay and by cell enumeration, as previously described.^49^

### Analyses of cell cycle and apoptosis

Cell cycle and apoptosis analyses were conducted as described previously.^49^ In brief, cells that had been exposed to GLPT at 0–2 mg/ml for 24–72 h were trypsinized and stained with a Cellular DNA Flow Cytometric Analysis Kit (for cell cycle progression) or an Annexin V-FITC Apoptosis Detection Kit I (for apoptosis) and then subjected to flow cytometry using a FACSCalibur flow cytometer (Becton Dickinson, San Jose, CA, USA). Cells treated with DMSO alone served as controls.

### Recombinant adenoviruses and infection of cells

The recombinant adenoviruses, including Ad-AMPKα-dn encoding c-myc-tagged dominant-negative AMPKα and Ad-GFP encoding GFP (green fluorescence protein), have been described previously.^50^ For experiments, A549 cells were infected with the appropriate adenoviruses for 24 h at a multiplicity of infection (MOI) of 1. Ad-GFP-infected cells served as controls.

### Western blotting

For western blotting, cells were briefly rinsed with ice-cold PBS (phosphate-buffered saline) and lysed. Then, the cell lysates were separated on an SDS-polyacrylamide gel, transferred to a PVDF membrane, and probed with the indicated antibodies, as described previously.^49^

### Statistical analysis

The results are presented as the mean ± SD (mean value ± standard deviation). Group variability and interactions were analyzed using one-way ANOVA, and Bonferroni’s posttests were used to compare replicate means. *P* < 0.05 was considered to indicate significance.
